# Group B Streptococcus antibiotics treatment during pregnancy and the risk of four neurodevelopmental outcomes in Swedish children

**DOI:** 10.1038/s41598-026-57533-y

**Published:** 2026-06-11

**Authors:** Unnur Gudnadottir, Sheila Orwa, Thi Cam Tu Ha, Martin J. Blaser, Sandra Guedes, Kelle Moley, Anders Elfvin, Kristin Wannerberger, Nele Brusselaers

**Affiliations:** 1 Department of Women’s and Children’s Health, Karolinska Institutet, Solna, 171 77 Sweden; 2 Global Health Institute, Department of Family Medicine and Population Health, University of Antwerp, Antwerp, 2610 Belgium; 3 Center for Advanced Biotechnology and Medicine, Rutgers University, New Brunswick, NJ USA; 4 Ferring Pharmaceuticals, Global Biometrics, Copenhagen, Denmark; 5 Department of Pediatrics, Institute of Clinical Sciences, Sahlgrenska Academy, University of Gothenburg, Gothenburg, Sweden; 6 Region Västra Götaland, Department of Pediatrics, Sahlgrenska University Hospital, Gothenburg, Sweden; 7 Ferring International Center SA, Ch de la Vergognausaz 50, Saint-Prex, 1162 Switzerland; 8 Department of Public Health and Primary Care, Ghent University, Ghent, 9000 Belgium

**Keywords:** Antibiotics, GBS, Intellectual disability, ADHD, Autism spectrum disorder, Cerebral palsy, Pregnancy, Diseases, Health care, Medical research, Neurology, Neuroscience

## Abstract

Recent studies have suggested an association between prenatal and early life antibiotics with adverse neurodevelopmental outcomes in children. As antibiotics during pregnancy are commonly prescribed, our aim was to estimate the association the groups of antibiotics prescribed for GBS during pregnancy the risk of neurodevelopmental outcomes in children. This population-based cohort study included all live births in Sweden from 2006 to 2016 (*n* = 1,066,777). Individual informed consent was not required, as the study used data from national registers and involved no direct participant contact. Using multivariable Cox Regression models and GEE models for secondary assessment we measured the association between seven first line treatment GBS antibiotics during pregnancy and (1) intellectual disability, (2) cerebral palsy, (3) autism spectrum disorder (ASD), and (4) attention deficit hyperactivity disorder (ADD/ADHD) as recorded in high-quality Swedish registers. GBS antibiotics were prescribed to 22.1% of mothers included. We found no association between GBS antibiotics and the risk of intellectual disability. However, a modest association was observed for cerebral palsy (aHR 1.17, 95%CI 1.06–1.29), ASD (aHR 1.12, 95%CI 1.07–1.17), and ADHD/ADD (aHR 1.17, 95%CI 1.12–1.22). This association was also seen when only including children born at term, and only children of mothers without autism spectrum disorder or ADHD diagnosis. The results were model dependent, as GEE models did not always show the same associations. Although the results could be affected by residual confounding or confounding by indication (as GBS infection has been associated with neurodevelopmental outcomes), they highlight the need for further studies on GBS antibiotic use during pregnancy, increased antibiotic stewardship with individual risk assessment and other treatment options for GBS, when needed.

**Supplementary Information:**

The online version contains supplementary material available at 10.1038/s41598-026-57533-y.

## Introduction

Globally 3% of children have intellectual disability, 0.2% have cerebral palsy, 1% have autism spectrum disorder (ASD) and 8% have attention deficit/hyperactivity disorder (ADD/ADHD)^1–4^, with comparable prevalences in Sweden^5–8^. These outcomes can have substantial impact on the quality of life of both children and their families. Although multicausal, emerging research suggests that neurodevelopmental conditions such as ASD and ADHD/ADD may be influenced by the gut microbiome via the gut-brain axis^9^.

In utero, fetuses are influenced by their mother’s microbiome via microbial products and microbially induced mechanisms including inflammation^10^. The microbiome of neonates is aquired at birth from mother via vertical transmission, including possible transmission of pathogens^11^. During the first 2–3 years of life the nervous system and gut microbiome undergo maturation where disturbances, such as antibiotics and infections, may have long-term effects^12–15^. Recent studies have found associations between both prenatal and early life antibiotic use and neurodevelopmental outcomes, where the first year of life is thought to be particularly important^16–20^. Furthermore, a recent Swedish study found associations between systemic antibiotic use during pregnancy and the risk of ASD and ADHD^9^.

Antibiotics are the most prescribed drugs during pregnancy, with an estimated 24% of women worldwide receiving antibiotics during their pregnancy^21^. Both genitourinary and respiratory infections are frequent during pregnancy, as well as group B Streptococcus (GBS) infection, with estimated 19.7 million pregnant women globally colonized in 2020^22^. Prevalence varies greatly by region^23^, and is estimated at 24.3–36% in Scandinavia in 2008^24^. GBS can cause maternal and neonatal harm ranging from infection to sepsis and stillbirth^23,25^. An estimated 1% of the newborns of colonized mothers develop early onset GBS (EOGBS) during the first six days of life^26^. In addition to prenatal antibiotic treatment for GBS, ~ 30% of pregnant women in high income countries receive intrapartum antibiotic prophylaxis (IAP) for early onset GBS infection^27^, which lowers the incidence of EOGBS^26,28,29^. Data on GBS treatment during pregnancy in Sweden is scarce, with one study from 2008 with a cohort of over 1,500 women, reporting 30% of women at risk (GBS positive during pregnancy, prolonged ruptured of membranes, preterm birth or temperature over 38 °C during delivery) receiving intraveneous intrapartum antibiotics^30^.

There is great need for studies on GBS antibiotics since they are so commonly used while their efficacy is debated due to rising resistance^31^, and their association with adverse childhood outcomes such as excessive weight gain^32^. Although antibiotics during pregnancy and early life has been associated with ASD, ADHD^9,17,19^, and intellectual disability^16,20^ focus on the common GBS specific antibiotics remains understudied.

Swedish population registers are widely used for their excellent population coverage and the possibility of linkages that allows investigation of rare outcomes, including neurodevelopmental outcomes^33^. Our aim was to assess the association between exposure to oral antibiotics used to treat GBS during pregnancy and the risk of the following neurodevelopmental outcomes in children; (1) intellectual disability, (2) cerebral palsy, (3) ASD, and (4) ADD/ADHD, using Swedish registers, with non-users of antibiotics as controls.

## Results

A total of 1,066,777 mother-child-dyads were studied, which included 710,332 individual mothers. Of those, 22.1% (235,803) were exposed to antibiotics used to treat GBS during their pregnancy (Table 1), and 2.0% (21,607) to possible IAP.

**Fig. 1:**
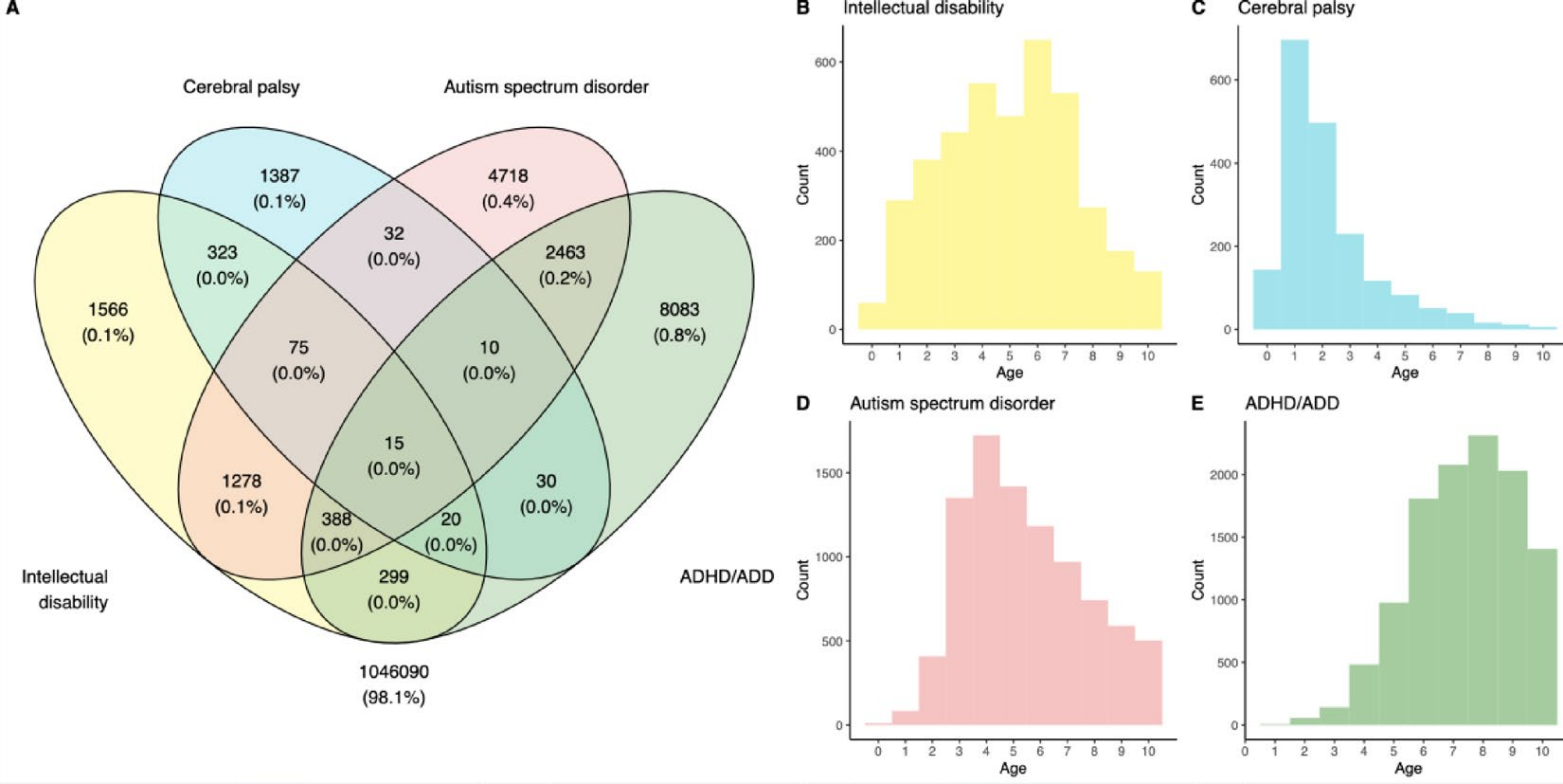
**(A)** Overview of the overlapping outcomes: Intellectual disability (yellow), cerebral palsy (blue), autism spectrum disorder (red), and ADHD/ADD (green). Distribution of age of first diagnosis for **(B)** intellectual disability, **(C)** cerebral palsy, **(D)** autism spectrum disorder, and (E) ADHD/ADD.

In total, 1.9% (20,687) of the children were diagnosed with any outcome of interest, of which 76.2% (15,754) had a single diagnosis and 23.8% (4,933) had multiple diagnoses (Fig. 1A). Altogether, 0.4% (3,964) of children were diagnosed with intellectual disability, 0.2% (1,892) with cerebral palsy, 0.8% (8,979) with ASD and 1.1% (11,308) with ADHD/ADD (Fig. 1A). The median follow-up age was 5 years (IQR 2–8). During the follow-up time, 2,803 children died (0.3%), most (2,028) during their first year of life. Cumulative incidence curves (**Figure ****S1**) show higher cumulative incidence for children exposed to GBS antibiotics compared to unexposed children.

### Intellectual disability

**Fig. 2:**
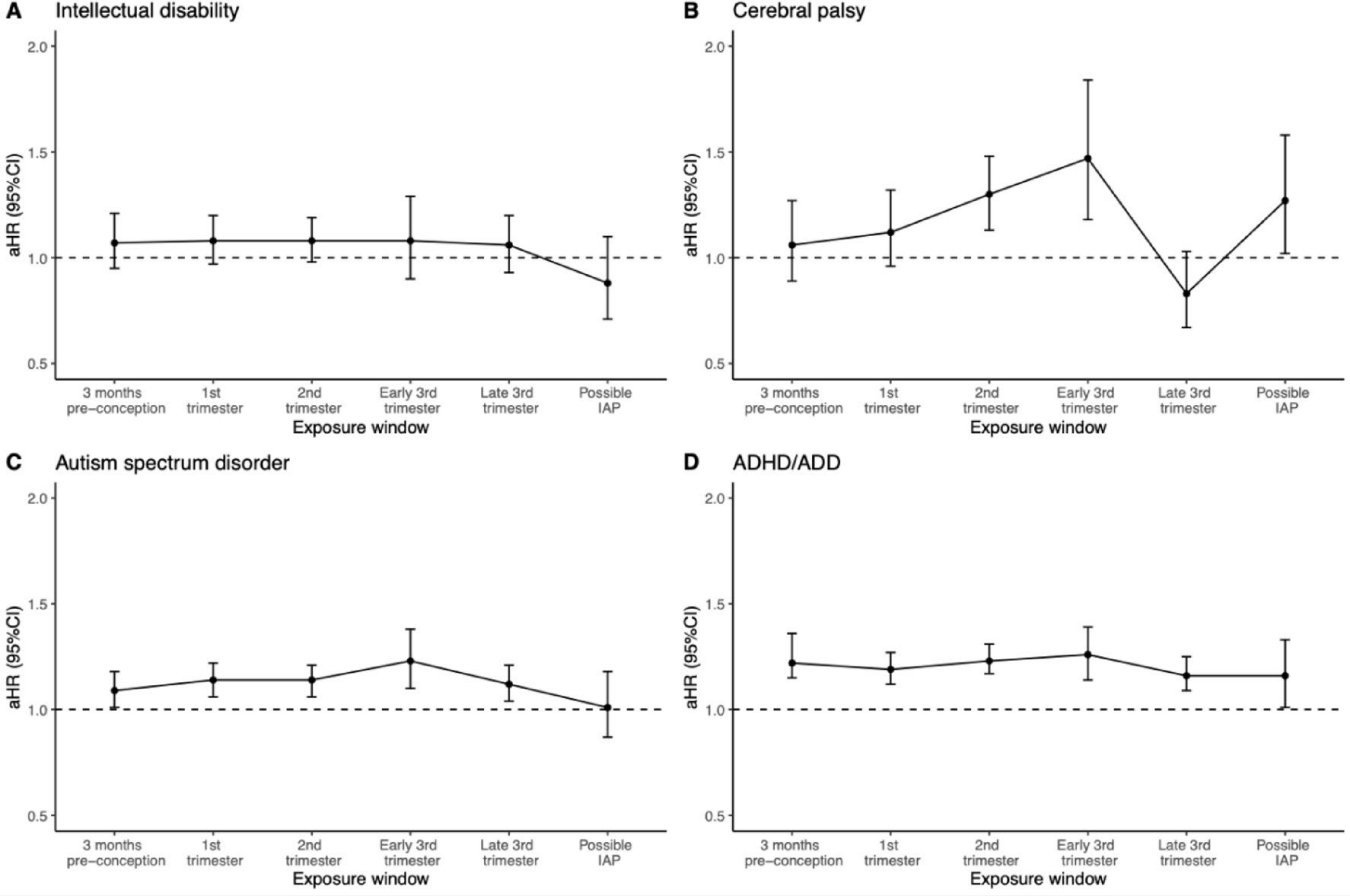
Adjusted hazard ratios (aHR) with 95% confidence intervals (CI) for **(A)** intellectual disability, **(B)** cerebral palsy, **(C)** autism spectrum disorder, and **(D)** ADHD/ADD for each exposure window. Reference are women without any antibiotic exposure during their entire pregnancy. The trimester-specific exposure groups were not mutually exclusive, and models were clustered by mother.

No association was found with GBS antibiotics and the risk of intellectual disability when investigating across the entire pregnancy and when divided by trimesters (Table 2, Table S1). No association was found with possible IAP and intellectual disability either (Fig. 2, Table S2). The potential confounders with the largest apparent effect (largest hazard ratios) were maternal ASD, being born preterm, being small for gestational age and having Apgar score < 7 at 5 min (all aHR > 2.0) (Table S1).

**Table 1:** Descriptive overview of the cohort, stratified by group B streptococcus (GBS) antibiotic exposure during pregnancy.

	Total	No systemic antibiotics	GBS antibiotics
	(*N* = 1,066,777)	(*N* = 830,974)	(*N* = 235,803)
Maternal age in years			
< 25	151,124 (14.2%)	113,485 (13.7%)	37,639 (16.0%)
25–29	317,465 (29.8%)	250,140 (30.1%)	67,325 (28.6%)
30–34	364,530 (34.2%)	286,708 (34.5%)	77,822 (33.0%)
> 34	233,658 (21.9%)	180,641 (21.7%)	53,017 (22.5%)
Maternal BMI (kg/m2)			
< 20.0	102,753 (9.6%)	80,328 (9.7%)	22,425 (9.5%)
20.0–24.9	515,140 (48.3%)	407,301 (49.0%)	107,839 (45.7%)
25.0–29.9	250,653 (23.5%)	193,854 (23.3%)	56,799 (24.1%)
> 30.0	126,242 (11.8%)	93,822 (11.3%)	32,420 (13.7%)
Missing	71,989 (6.7%)	55,669 (6.7%)	16,320 (6.9%)
Parity			
Multiparous	597,477 (56.0%)	455,041 (54.8%)	142,436 (60.4%)
Nulliparous	469,300 (44.0%)	375,933 (45.2%)	93,367 (39.6%)
Nordic country of birth			
Yes	818,784 (76.8%)	638,510 (76.8%)	180,274 (76.5%)
No	247,993 (23.2%)	192,464 (23.2%)	55,529 (23.5%)
Cohabiting			
Living with father	955,095 (89.5%)	747,239 (89.9%)	207,856 (88.1%)
Not living with father	111,672 (10.5%)	83,727 (10.1%)	27,945 (11.9%)
**Maternal health characteristics**			
Tobacco use	73,332 (6.9%)	52,165 (6.3%)	21,167 (9.0%)
Epilepsy	5,410 (0.5%)	3,955 (0.5%)	1,455 (0.6%)
Asthma	77,418 (7.3%)	55,807 (6.7%)	21,611 (9.2%)
Hypertension	5,115 (0.5%)	3,736 (0.4%)	1,379 (0.6%)
Diabetes mellitus or gestational diabetes	22,036 (2.1%)	15,439 (1.9%)	6,597 (2.8%)
Hypothyroidism	34,159 (3.2%)	24,870 (3.0%)	9,289 (3.9%)
Hyperthyroidism	6,570 (0.6%)	4,875 (0.6%)	1,695 (0.7%)
Autism spectrum disorder (ASD)	3,581 (0.3%)	2,442 (0.3%)	1,139 (0.5%)
ADHD/ADD	18,994 (1.8%)	12,426 (1.5%)	6,568 (2.8%)
Prescribed NSAIDs during pregnancy	35,277 (3.3%)	23,130 (2.8%)	12,147 (5.2%)
Prescribed PPIs during pregnancy	35,611 (3.3%)	23,178 (2.8%)	12,433 (5.3%)
Analgesics 3 months before or during pregnancy	308 (0.0%)	203 (0.0%)	105 (0.0%)
Antiepileptics 3 months before or during pregnancy	9,381 (0.9%)	6,363 (0.8%)	3,018 (1.3%)
Psycholeptics 3 months before or during pregnancy	58,587 (5.5%)	39,419 (4.7%)	19,168 (8.1%)
Phsycoanaleptics 3 months before or during pregnancy	71,037 (6.7%)	49,647 (6.0%)	21,390 (9.1%)
**Pregnancy/birth characteristics**			
Assisted conception			
Yes	30,956 (2.9%)	24,315 (2.9%)	6,641 (2.8%)
No	1,035,821 (97.1%)	806,659 (97.1%)	229,162 (97.2%)
Delivery mode			
Vaginal delivery	896,247 (84.0%)	703,590 (84.7%)	192,657 (81.7%)
Elective C-section	75,670 (7.1%)	55,906 (6.7%)	197,64 (8.4%)
Emergency C-section	94,850 (8.9%)	71,470 (8.6%)	23,380 (9.9%)
Sex of child			
Boy	548,769 (51.4%)	427,259 (51.4%)	121,510 (51.5%)
Girl	518,007 (48.6%)	403,714 (48.6%)	114,293 (48.5%)
Preterm	50,154 (4.7%)	37,085 (4.5%)	13,069 (5.5%)
Large for gestational age	36,926 (3.5%)	27,386 (3.3%)	9,540 (4.0%)
Small for gestational age	24,256 (2.3%)	18,879 (2.3%)	5,377 (2.3%)
Apgar score < 7 at 5 min	12,674 (1.2%)	9,334 (1.1%)	3,340 (1.4%)
**Outcomes of interest**			
Child intellectual disability	3,964 (0.4%)	2,933 (0.4%)	1,031 (0.4%)
Child cerebral palsy	1,892 (0.2%)	1,370 (0.2%)	522 (0.2%)
Child autism spectrum disorder (ASD)	8,979 (0.8%)	6,547 (0.8%)	2,432 (1.0%)
Child ADHD/ADD	11,308 (1.1%)	7,891 (0.9%)	3,417 (1.4%)

Group B streptococcus (GBS), body mass index (BMI), attention deficit/hyperactivity disorder (ADHD/ADD), nonsteroidal anti-inflammatory drug (NSAID), proton pump inhibitor (PPI), Caesarean section (C-section).

**Table 2:** Multivariable Cox models, with group B streptococcal (GBS) antibiotics during pregnancy as exposure and women not exposed to any systemic antibiotics during pregnancy as reference. Results reported as adjusted hazard ratios (aHR) with 95% confidence intervals (CI). Significant values formatted in bold. No HR were generated for groups with less than 40 cases.

	Intellectual disability		Cerebral palsy		Autism spectrum disorder		ADHD/ADD	
	*n*	aHR (95% CI)	*n*	aHR (95% CI)	*n*	aHR (95% CI)	*n*	aHR (95% CI)
**All pregnancies**	3,964	1.07 (0.99–1.15)	1,892	**1**.**17 (1**.**06–1**.**29)**	8,979	**1**.**12 (1**.**07–1**.**17)**	11,31	**1**.**17 (1**.**12–1**.**22)**
**Delivery mode**								
Vaginal delivery	2,969	1.07 (0.99–1.16)	1,208	1.12 (0.99–1.28)	6,997	**1**.**10 (1**.**04–1**.**16)**	9,129	**1**.**16 (1**.**11–1**.**22)**
Elective C-section	336	1.02 (0.80–1.29)	145	0.98 (0.69–1.41)	784	1.15 (0.99–1.34)	895	**1**.**19 (1**.**03–1**.**37)**
Emergency C-section	659	1.12 (0.95–1.33)	539	**1**.**31 (1**.**09–1**.**57)**	1,198	**1**.**24 (1**.**10–1**.**41)**	1,284	**1**.**20 (1**.**06–1**.**35)**
**Preterm status**								
Only preterm birth	556	**1**.**28 (1**.**07–1**.**53)**	572	**1**.**31 (1**.**10–1**.**56)**	714	**1**.**38 (1**.**18–1**.**61)**	818	**1**.**34 (1**.**15–1**.**56)**
Only term birth	3,408	1.05 (0.97–1.14)	1,320	**1**.**13 (1**.**00–1**.**28)**	8,265	**1**.**10 (1**.**05–1**.**16)**	10,490	**1**.**16 (1**.**11–1**.**21)**
**Maternal neurological diagnoses**								
Only mothers with autism spectrum disorder (ASD)	44	0.76 (0.37–1.56)	13	**-**	251	1.12 (0.85–1.47)	236	**1**.**35 (1**.**01–1**.**79)**
Only mothers with ADHD/ADD	113	1.24 (0.85–1.82)	45	0.74 (0.40–1.39)	593	1.09 (0.91–1.29)	1,345	1.07 (0.95–1.20)
Only mothers without autism spectrum disorder (ASD) and ADHD/ADD	3,824	**1**.**08 (1**.**01–1**.**16)**	1,838	**1**.**19 (1**.**07–1**.**32)**	8,285	**1**.**12 (1**.**07–1**.**18)**	9,890	**1**.**17 (1**.**12–1**.**22)**

Adjusted hazard ratio (aHR), confidence interval (CI), Caesarean section (C-section), attention deficit/hyperactivity disorder (ADHD/ADD).

The proportional Hazard assumption was met for the final reduced model (global *p* = 0.07) when stratifying for maternal BMI, and Schoenfield residual plots showed no pattern with time. Sensitivity analysis showed similar results. GEE models showed similar results, and no multicollinearity was detected with the variables included in the model.

### Cerebral palsy

Exposure to GBS antibiotics during any time during pregnancy was associated with an increased risk of cerebral palsy (aHR 1.17, 95%CI 1.06–1.29) (Table 2, Table S1). The potential confounders that appeared to have the largest effect were being born preterm and having Apgar < 7 at 5 min (both aHR ≥ 2.0) (Table S1).The proportional Hazard assumption was met for the final reduced model (global *p* = 0.34) when stratifying for Apgar score at 5 min (above or below 7), and Schoenfield residual plots showed no pattern with time. Sensitivity analysis showed similar results.

When timing of exposure was investigated, an association was found with GBS antibiotics in second (aHR 1.30, 95%CI 1.14–1.48) and the early third (aHR 1.47, 95%CI 1.18–1.84) trimester. An association also was found between possible IAP and increased risk of cerebral palsy (aHR 1.27, 95%CI 1.02–1.58) (Fig. 2, Table S2).

A GEE model found no association between GBS antibiotics and cerebral palsy (*p* = 0.46). The discrepancy with the other results could be due to the GEE model not being sensitive to time-varying effects, or being underpowered as only children who reached the age of 10 were included. No multicollinearity was found with the included variables.

### Autism spectrum disorder

An association was found between GBS exposure during pregnancy and increased risk of ASD (aHR 1.12, 95%CI 1.07–1.17) (Table 2, Table S1). The potential confounder that appeared to have the largest effect was maternal ADHD/ADD (aHR 4.10) (Table S1). The final reduced model was stratified by maternal ADHD/ADD diagnosis and country of birth to account for violations of the proportional hazard assumption. Maternal tobacco use during pregnancy was included as a time-dependent covariate for the same reason. Sensitivity analysis showed similar results.

Similar associations were found for all exposure windows, being the highest in early third trimester (aHR 1.23, 95%CI 1.10–1.38). No association was found between possible IAP and the risk of autism spectrum disorder (Fig. 2, Table S2).

A GEE model (using the same variables, but without stratification or time dependent variables), found no association between GBS antibiotics and ASD (*p* = 0.12). The discrepancy with the other results could be as described above for cerebral palsy. No multicollinearity was detected.

### ADHD/ADD

An association was found between GBS antibiotic use during pregnancy and increased risk of ADHD/ADD (aHR 1.17, 95%CI 1.12–1.22) (Table 2, Table S1).The potential confounders that appeared to have the largest effect were maternal age < 25 years, maternal BMI > 30, tobacco use, maternal ADHD/ADD and being small for gestational age (all aHR > 1.5) (Table S1).The final model was reduced via backward selection and stratified by maternal ASD diagnosis and country of birth to account for violations of the proportional hazard assumption. Maternal tobacco use during pregnancy was included as a time-dependent covariate for the same reason. Sensitivity analysis showed similar results.

The same association was seen for all exposure windows, being the highest in the early third trimester (aHR 1.26, 95%CI 1.14–1.39). An association also was found between possible IAP and ADHD/ADD (aHR 1.16, 95%CI 1.01–1.33) (Fig. 2, Table S2). Furthermore, in a GEE model (using the same variables, but without stratification or time dependent variables), an association was found between GBS antibiotic exposure during pregnancy and ADHD/ADD (*p* = 0.00000005). No multicollinearity was found.

### Stratified analysis

#### Intellectual disability

No association was found between GBS antibiotics at any time during pregnancy and the risk of intellectual disability for any delivery mode. However, an association was found when only examining children born prematurely (aHR 1.28, 95%CI 1.07–1.53) and only examining women who were not diagnosed with ASD or ADHD/ADD (aHR 1.08, 95%CI 1.01–1.16) (Table 2).

#### Cerebral palsy

An association was found between GBS antibiotics at any time during pregnancy and the risk of cerebral palsy for children born via emergency C-section (aHR 1.31, 95%CI 1.09–1.57). When stratifying based on preterm status, an association was found for both children born prematurely (aHR 1.31, 95%CI 1.10–1.56) and children born at term (aHR 1.13, 95%CI 1.00–1.28). When stratifying for maternal neurological diagnoses, an association was found when only including women without ASD or ADHD/ADD diagnosis (aHR 1.19, 95%CI 1.07–1.32) (Table 2).

#### Autism spectrum disorder

An association was found between GBS antibiotics at any time during pregnancy and the risk of ASD for all delivery modes (vaginal: aHR 1.10, 95%CI 1.04–1.16, elective C-section: aHR 1.16, 95%CI 1.00–1.35, emergency C-section: aHR 1.24, 95%CI 1.10–1.41). Furthermore, GBS antibiotics were associated with an increased risk of ASD both for children born prematurely (aHR 1.37, 95%CI 1.17–1.60) and children born at term (aHR 1.10, 95%CI 1.05–1.16). When stratifying for maternal neurological diagnoses, an association was found when only including women without ASD or ADHD/ADD (aHR 1.12, 95%CI 1.07–1.18) (Table 2).

#### ADHD/ADD

An association was found between GBS antibiotics at any time during pregnancy and the risk of ADHD/ADD for all delivery modes (vaginal: aHR 1.16, 95%CI 1.11–1.22, elective C-section: aHR 1.19, 95%CI 1.04–1.37, emergency C-section: aHR 1.20, 1.06–1.35). An association also was found both for children born prematurely (aHR 1.34, 95%CI 1.16–1.55) and children born at term (aHR 1.16, 95%CI 1.11–1.21). When stratified by maternal neurological diagnoses, an association was found both when only looking at mothers diagnosed with ASD (aHR 1.35, 95%CI 1.01–1.79) and mothers without diagnosis of ASD or ADHD/ADD (aHR 1.17, 95%CI 1.12–1.22) (Table 2).

### Dose response analysis

The average DDD for all outcomes was an estimated 14 days of exposure, and median 10 days with IQR 6–14.

**Fig. 3:**
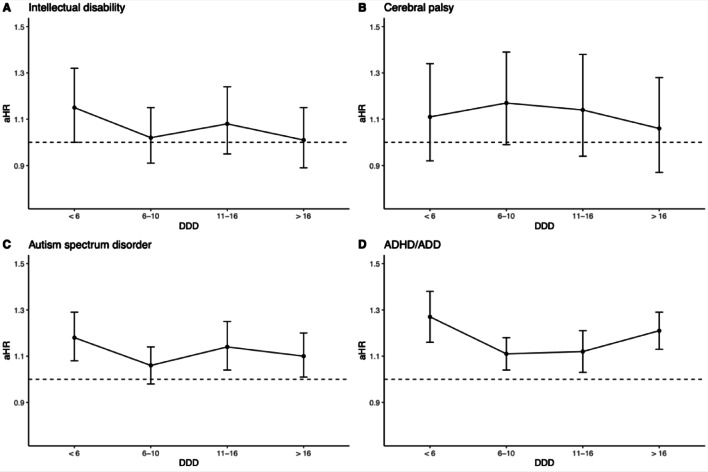
Dose response analysis for GBS antibiotics at any time during pregnancy, as defined daily dose (DDD) quartiles for **(A)** intellectual disability, **(B)** cerebral palsy, **(C)** autism spectrum disorder and **(D)** ADHD/ADD. No GBS antibiotics during pregnancy was the reference in all analyses.

For intellectual disability, an association was found between DDD < 6 during pregnancy and increased risk of intellectual disability (aHR 1.15, 95%CI 1.00–1.32) (Fig. 3A). No association was found for any DDD of GBS antibiotics and the risk of cerebral palsy (Fig. 3B). For both ASD (Fig. 3C) and ADHD/ADD (Fig. 3D), the risk was highest for the lowest DDD quartile (< 6) (ASD : aHR 1.18, 95%CI 1.08–1.29, ADHD/ADD: aHR 1.27, 95%CI 1.16–1.38).

When considering number of prescriptions, one prescription at any time during pregnancy was sufficient for an increased risk of cerebral palsy (Figure S2B), ASD (Figure S2C) or ADHD/ADD (Figure S2D).

## Discussion

In this Swedish population-based cohort study we found a modest association between women who were exposed to GBS antibiotics during pregnancy and their children being diagnosed with cerebral palsy, ASD or ADHD/ADD in early childhood. These results could not be fully explained by preterm birth, a well-known risk factor, as they were also observed in children born at term, but delivery mode and maternal neurological diagnoses seemed important confounders. Furthermore, we found that timing of exposure seemed to affect the risk of cerebral palsy and ASD, but not ADHD/ADD. No clear dose-response was observed. However, the results were model dependent as the same association was not always found when using GEE models. This could be due to the fact that the GEE models only included children that either had the outcome of interest or had turned 10 years old but not the whole population, or because they do not include time-to-event as a variable as the Cox models do.

In all models the maternal country of birth had an effect on the results, which could be due to mothers born in Sweden (or other Nordic countries) being more familiar with the Swedish healthcare system, more likely to seek diagnoses for their children, or due to some cultural biases. As expected, being born prematurely, having low Apgar score, maternal neurological outcomes and sex of the child (with girls having a lower risk) were important confounders. Additionally, when our models were restricted to children born prematurely, an association was found between GBS antibiotics and intellectual disabilities – but as that was the only model in which an association was found with intellectual disabilities it may be that the preterm status is the main contributing factor. For our other outcomes of interest, an association continued to be observed when only including children born at term. More importantly, when restricting to mothers without diagnosed ASD or ADHD/ADD (to avoid predisposition/heritability effects), we found that prenatal GBS antibiotic exposure during pregnancy was associated with an increased risk of all four neurodevelopmental outcomes in children. Both of these stratified models support our hypothesis that GBS antibiotics play a part in neurodevelopmental outcomes, as the two major known predisposing risk factors (being born preterm, and maternal history of ASD or ADHD/ADD) are excluded. The dose response results for all outcomes were non-linear and warrant cautious interpretation due to potential confounding by indication, uneven number of women in each group (as most only got one prescription) or dose truncation. Furthermore, the low number of women who received ≥3 prescriptions also lead to broad confidence intervals, and their interpretation should therefore be interpreted with caution.

A major strength of this study is the use of population-based registers, as antibiotics are only available after prescription in Sweden. We can therefore expect a low risk of exposure misclassification, except for possible non-compliance with intake/completion of dose, although a recent Swedish study showed high compliance (98%) of antibiotic intake during pregnancy^34^. Furthermore, our use of multiple registers minimizes the risk of recall bias, loss to follow-up, and volunteer bias.

The main weakness of the study is confounding by indication, and that we do not have data if the antibiotics were indeed used to treat GBS or some other infection. Indications are not recorded in the Swedish Prescribed Drugs Registry, and the In- and Out-Patient Registry does not adequately capture indications and omits primary care diagnosis. The antibiotics included as exposures are the first line of treatment for GBS during pregnancy in Sweden and were the exposure of interest for this study, and not the GBS infection itself. Other infections during pregnancy may confound our results, and future studies should therefore aim to include the underlying cause for antibiotics in their models. There is also a risk of misclassification for IAP, as the variable was inferred from other diagnostic codes. There is also possible residual confounding, as data on maternal infection severity, socioeconomic status and health seeking behaviour was not available. Additional factors not included in this study such as asphyxia, brain pathology and genetic syndromes could also impact the risk of some of the investigated outcomes. Furthermore, some of the outcomes estimated may have delayed diagnosis and we would therefore have some misclassification (false negatives), especially in girls^35^. This would especially be relevant for multifactorial outcomes that are on a spectrum, such as ASD and ADHD/ADD. The outcomes of interest are most likely all multicausal where parental health, genetic and environmental factors play a role^36^. We speculate that there may be an underlying genotype and/or predisposition that could have different expression depending on external factors such as the microbiome, as has been suggested for other neurological outcomes^18,37^. Our data also did not include information on neurodevelopmental conditions of the fathers, which could influence the genetic risk of our outcomes of interest. Lastly, results from the GEE models were not always consistent with the Cox models, which could be due to the different nature of the models and the questions they aim to answer. More specifically, it could indicate that the exposure influences the timing of the outcome but not the overall outcome across timepoints or that it is not sensitive to time-varying effects as Cox models include time-to-event as a variable while GEE models do not. It should also be noted that as this is an open cohort, some children may be too young to be diagnosed with the outcomes of interest at the end of the study. Lastly, our results may not be generalizable as other countries may have other GBS treatment strategies.

Our results are consistent with previous studies that have focused on all systemic antibiotics, while we isolated antibiotics used to treat GBS (independent of the indication)^9,19^. This study, via design, cannot show any causal relationship or mechanistic insight between GBS treatment and neurodevelopmental outcomes due to its observational nature. However, it is possible to hypothesize that the antibiotics used to treat GBS may reach the children through chord blood in utero, via breastmilk^38^, or have effects due to suboptimal acquisition and/or early maturation of the microbiome^13^. The suboptimal (dysbiotic) microbiome may then influence the risk of neurodevelopmental outcomes via the gut-brain axis at a time in the child’s life in which neuroplasticity is high^14^.

One of the main reasons for administering GBS-treating antibiotics during pregnancy is to minimize the risk of transmission to the infant. Studies have shown that approximately 1% of mother’s transfer GBS to their infant in it’s first days of life^27^, but risks that have been found to be associated with prenatal antibiotic exposure both in the current and previous studies^9,19,39^. Antibiotics are often lifesaving and necessary for mother and child. After further studies and possible development of alternative treatments, it may be beneficial to examine either a more individual-based approach of when to administer antibiotics taking clinical trade-offs into account, or to consider other means of preventing neonatal GBS infection, such as non-antibiotic treatments or maternal vaccination^25^. Furthermore, it must be noted that as confounding by indication cannot be excluded, as GBS has previously been associated with neurodevelopmental outcomes^40^. More research is needed, particularly for children with an a priori increased risk of neurodevelopmental issues and delays in development.

To conclude, this study on the Swedish pregnant population shows a modest association between GBS antibiotics and cerebral palsy, ASD and ADHD/ADD in children when using multivariable Cox regression models. For all three outcomes, a single prescription during pregnancy was associated with increased risk, but our analysis separated into trimesters also indicate that the risk varies by timing of exposure. Our findings should further be validated in other populations, as prescription rates and other environmental risk factors vary globally. Furthermore, studies including the indiciation for drug use would be useful to provide further clarity to the findings.

## Methods

### Aim

The aim of this study was to assess the association between oral antibiotics used to treat prenatal GBS and four neurodevelopmental outcomes in children; (1) intellectual disability, (2) cerebral palsy, (3) ASD, and (4) ADD/ADHD.

### Study design and period

For this cohort study, data on all births in Sweden from July 1 st 2006 – December 31 st 2016 was extracted from high quality national registers: the Medical Birth Register (established in 1973)^41^, the Swedish Patient Register (full nationwide coverage since 1987)^42^, the Swedish Cause of Death Register (established 1952)^43^, and the Swedish Prescribed Drug Register (established in 2005)^33^, as published previously^44^. Data were linked using Swedish personal identity numbers. This study was performed in accordance with the Declaration of Helsinki, and STROBE (Supplementary material), and with ethical approval (2017/2423-31) from the Swedish Ethical Review Authority. Informed consent was not required by Swedish Ethical Review Authority (as mentioned is noted in the ethical approval) as this study is based on Swedish national registers and no involvement of patients was needed. Gender of the mothers was not recorded in the dataset, but for consistency we refer to the biological sex of pregnant individuals as “women/mothers” throughout this manuscript.

### Inclusion and exclusion criteria

Only liveborn singleton births were included in the analysis (*n* = 1,095,644, *n* = 1,066,777 after exclusion). Women exposed to GBS antibiotics with or without other systemic antibiotics (*n* = 235,803), or completely antibiotic-unexposed (*n* = 830,974) were included in this study. Women who were only exposed to antibiotics that are not used for GBS treatment/prophylaxis were excluded from the study (*n* = 28,867).

### Exposure

The study exposure was defined as at least one dispensed prescription (oral) antibiotics traditionally used to treat GBS in the Prescribed Drug Registry^27^. Drug prescriptions were recorded according to World Health Organization (WHO) Anatomical Therapeutic Chemical (ATC) classifications^45^; primary treatment penicillin (ATC: J01C), alternative for those with allergies are other beta-lactams (ATC: J01DB), cefazolin (ATC: J01DB04), vancomycin (ATC: A07AA09/J01XA01), clindamycin (ATC: J01FF), erythromycin (ATC: J01FA01) or nitrofurantoin (ATC: J01XE01)^27,46^. Participants who did not receive any systemic antibiotics during their pregnancy (whether GBS-aimed or not) were used as the reference group.

At the time of data extraction, Sweden employed a risk-based strategy to decide whether to prescribe IAP; it was reserved for women who had a positive GBS test at the end of their pregnancy (week 35–37), or those with preterm birth (before 37 weeks), or during prolonged rupture of membranes and intrapartum fever. Therefore, as we do not have access to data on the drugs received in hospital, those that had diagnosis codes O23.4 (GBS detected in urine during pregnancy), O42.1 (prolonged rupture of membranes (> 18 h)), O75.2 (intrapartum fever *≥* 38 °C) and those that gave birth preterm were considered as having received IAP in this study^47^. Furthermore, asymptomatic GBS is not treated in Sweden at time of data extraction^46^. Data on possible IAP was used in a separate model from data on exposure during pregnancy.

The accumulated defined daily doses (assumed average dose per day, DDD), number and timing of prescriptions was recorded. Five exposure windows were used: (1) pre-conception (90 days before conception), (2) first trimester (0–97 days of gestation), (3) second trimester (98–202 days of gestation), (4) early third trimester (203–223 days of gestation), and (5) late third trimester (224 days – delivery). Date of last menstrual period was collected during early pregnancy midwife check-ups. As the date of birth was provided in year/month format due to Swedish privacy regulations, it was set as the 15th of each month in all of the observations. These exposure windows were calculated using the date of the last menstrual period.

### Outcomes

Four neurodevelopmental disorders were assessed using the In- and outpatient registry with ICD-10 codes: Intellectual disability (Psychological development disorders, F70-79), Autism spectrum disorder (F84), ADHD/ADD (F90) and Cerebral palsy (G80). The groups are not mutually exclusive.

### Covariates

Other extracted covariates from the Swedish Medical Birth Register are: (1) maternal factors (age at childbirth (as groups: ≤25, 25–29, 30–34, 35–39, ≥ 40 years), country of birth (Nordic or non-Nordic), body mass index (BMI, as groups: < 20.0, 20.0–24.9, 25.0–29.9, ≥ 30.0 kg/m^2^, or missing), parity (nulliparous or not), and use of artificial reproductive technologies (ART), (2) maternal lifestyle factors (drug use during pregnancy (proton pump inhibitors (PPIs), nonsteroidal anti-inflammatory drugs (NSAIDs), and neurological medication (analgesics (ATC: N02), antiepileptics (ATC: N03), psycholeptics (ATC: N05) or psychoanaleptics (ATC: N06)) 3 months prior to or during pregnancy, any tobacco consumption during pregnancy (smoking and smokeless tobacco)), (3) common maternal chronic comorbidities (separately: asthma, epilepsy, hypertension, diabetes, hypo- and hyperthyroidism, autism spectrum disorder and ADHD), and (4) child related factors (sex of the child, size [small (< 10th percentile), appropriate, large (> 90th percentile) for gestational age], delivery mode (vaginal, elective- or emergency C-section, Apgar score at 5 min (lower or higher than 7) and gestational age [preterm (birth before 37 completed gestational weeks)] or not.

### Statistical analysis

The risk of adverse neurological outcomes after GBS treatment exposure (at any time and for each exposure window) was calculated using multivariable Cox regression models^48^, taking into account clustering by mother. All children were followed from birth until first diagnosis, end of the study (December 2017) or death, whichever came first.

The models were adjusted for potential maternal, obstetric and neonatal confounders (listed above) using backward selection (using *p* < 0.05 threshold), along with the pre-selected variables maternal age, maternal BMI and sex of child. Results were presented as adjusted hazard ratio (aHR) with 95% confidence intervals (CI). The proportional hazard assumption of the models was tested using Schoenfeld residuals and time-dependent covariate analysis. In the case of models not passing the assumption, the Cox models were either stratified and/or included as time-varying covariates using the formula function(x, t, …) x * log(t). First the variables with the largest effects were stratified for, and if that was not enough to meet the assumptions, then covariates were included as time-varying.”

For comparison with the Cox models, Generalized estimating equation (GEE) models were used to account for mothers with multiple pregnancies during the study period including only children with the outcome, or those that had reached the age of 10. Children that had neither reached the age of 10 nor had any outcome were excluded from this analysis. Results from GEE models were reported as finding an association (*p* < 0.05) or not (*p* > 0.05).

Unadjusted cumulative incidence curves were produced, and dose response analysis was conducted using both DDD and number of prescriptions (1, 2, or ≥3). Stratified analysis was performed for delivery mode (vaginal, elective C-section, emergency C-section), preterm status (only children born preterm, only children born at term) and neurological diagnoses of the mother (ASD, ADHD/ADD).

Absence of reporting of the respective ICD codes was considered absence of the disease/disorder. If a substantial group of individuals had missingness on a certain variable (e.g., BMI is missing in 7% in this cohort), a dummy variable was created to keep the individuals in the models. Sensitivity analyses were performed, excluding those with missing BMI. For other variables, missingness was limited and considered as absence/No for variables where appropriate.

RStudio (version 2023.12.1 + 402) was used for all statistical analyses and ggplot2 (v. 3.5.0) and ggvenn (0.1.10) to create figures^49–51^. The packages survival (v. 3.7.0) and survminer (v. 0.4.9) were used for Cox modelling^48,52^.

## Supplementary Information

Below is the link to the electronic supplementary material.

Supplementary Material 1

## Data Availability

Individual participant data can not be made available to others, due to the personal information found there. The data dictionary, study protocol and the statistical analysis plan can be made available upon reasonable request to the corresponding author.

## Abbreviations

ADD/ADHD: attention deficit/hyperactivity disorder
ART: artificial reproductive technologies
ASD: autism spectrum disorder
ATC: Anatomical Therapeutic Chemical
BMI: body mass index
DDD: defined daily doses
EOGBS: early onset GBS
GBS: group B Streptococcus
GEE: Generalized estimating equation
IAP: intrapartum antibiotic prophylaxis
NSAIDs: nonsteroidal anti-inflammatory drugs
PPIs: proton pump inhibitors
WHO: World Health Organization
