# Supplementary material for: Group B Streptococcus antibiotics treatment during pregnancy and the risk of four neurodevelopmental outcomes in Swedish children
Source: Sci Rep. 2026 Jun 11;16:18192. doi: 10.1038/s41598-026-57533-y (PMC13260725; doi:10.1038/s41598-026-57533-y)
Supplement: Supplementary file 1 — Supplementary Material 1 [file 41598_2026_57533_MOESM1_ESM.pdf]

Supplementary Material

Table of contents

Figure S1: Unadjusted cumulative incidence curves

Figure S2: Dose response analysis considering number of prescriptions

Table S1: Multivariable Cox results

Table S2: Stratification by trimester

STROBE Checklist

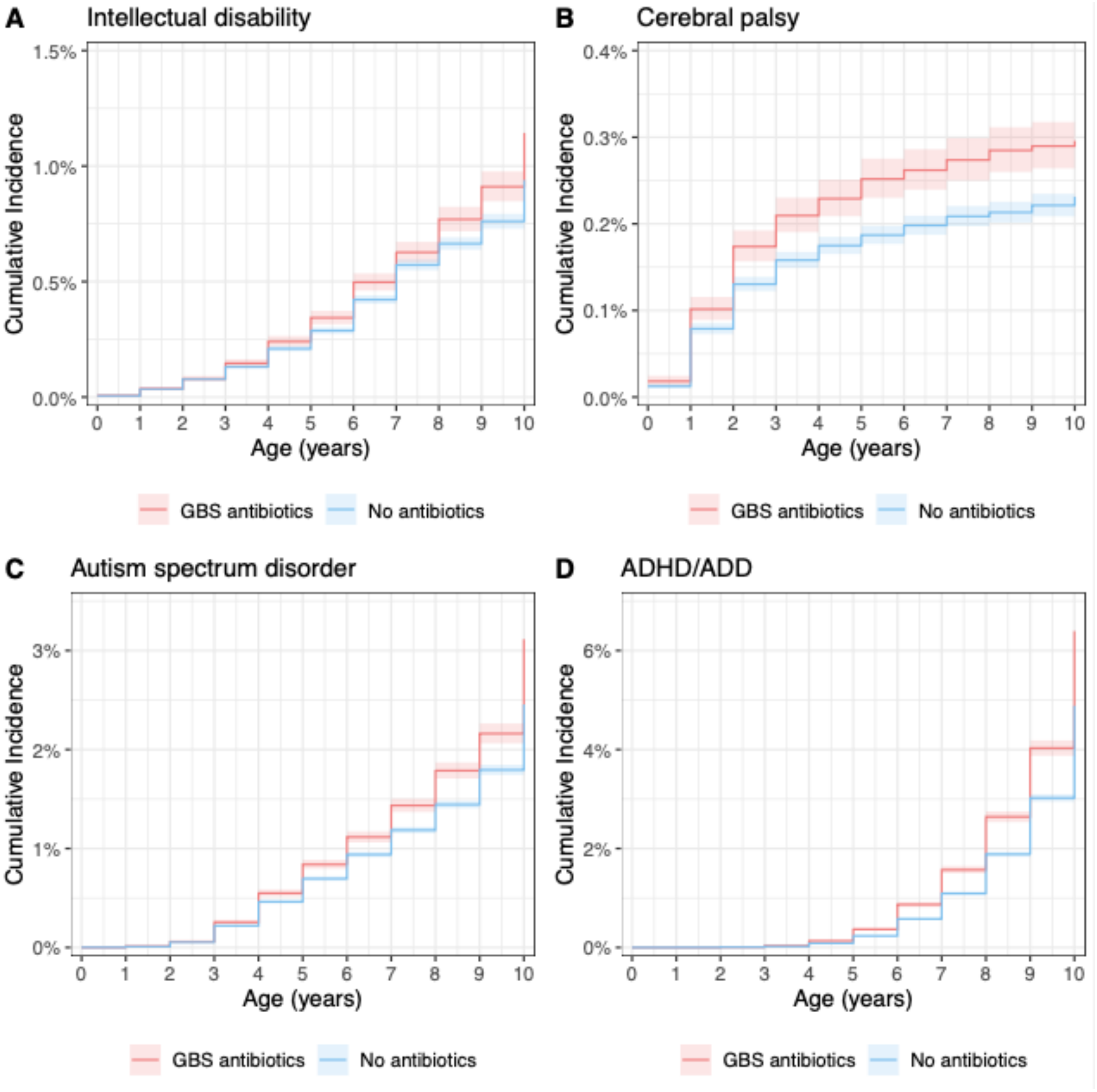

Figure S1: Unadjusted cumulative incidence curves for GBS antibiotics or no antibiotics and A) intellectual disability, B) cerebral palsy, C) autism spectrum disorder, and D) ADHD/ADD.

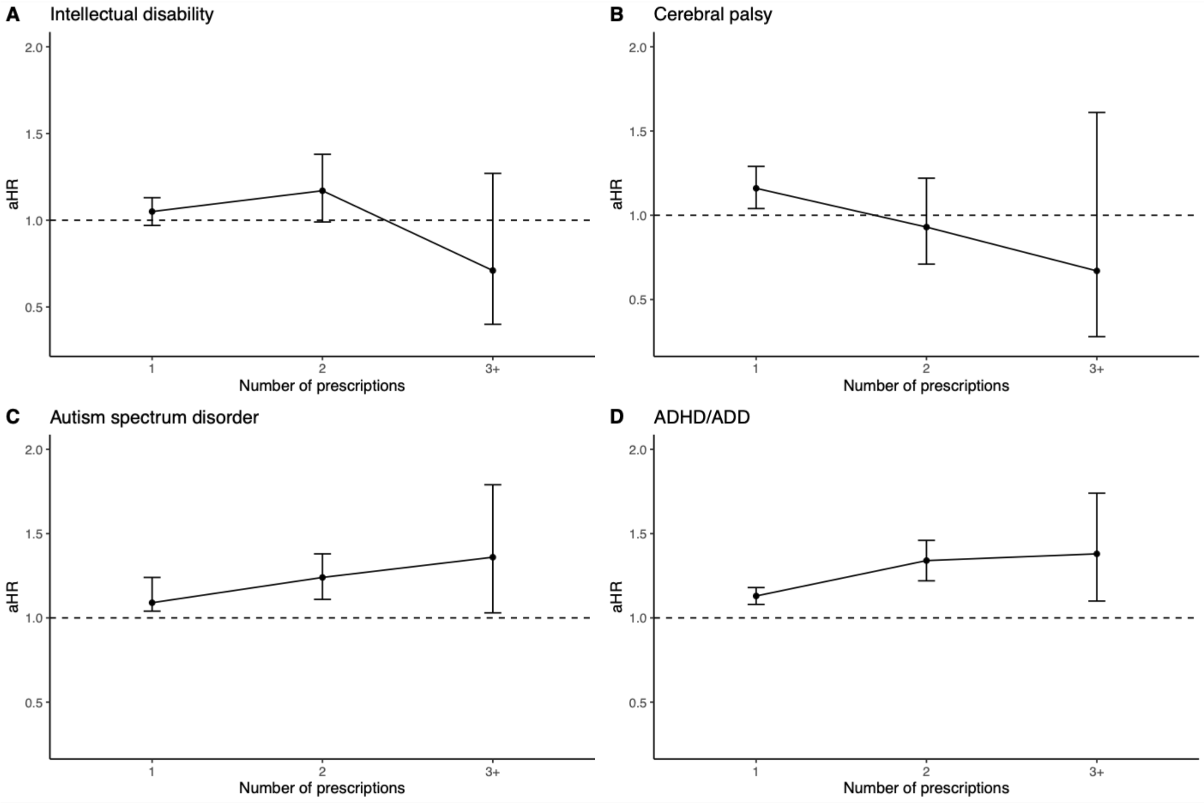

Figure S2: Dose response analysis for GBS antibiotics at any time during pregnancy, as defined daily dose (DDD) quartiles for A) intellectual disability, B) cerebral palsy, C) autism spectrum disorder and D) ADHD/ADD. No GBS antibiotics during pregnancy as reference in all analyses.

Table S1: Results from reduced multivariable Cox regression models for the four outcomes of interest as adjusted hazard ratios (aHR) and 95% confidence intervals (CI). Significant results formatted in bold. Variables which were included as stratified or time dependent (function(x, t, …) x * log(t)) in the models are reported as such.

	**Intellectual disability** aHR (95% CI)	**Cerebral palsy** aHR (95% CI)	**Autism spectrum disorder** aHR (95% CI)	**ADHD/ADD** aHR (95% CI)
GBS antibiotics any time during pregnancy	1.07 (0.99-1.15)	**1.17 (1.06-1.29)**	**1.12 (1.07-1.17)**	**1.17 (1.12-1.22)**
Maternal age in years				
< 25	**0.90 (0.81-0.99)**	1.10 (0.95-1.28)	**0.89 (0.83-0.96)**	**1.62 (1.52-1.72)**
25 - 29	REF	REF	REF	REF
30 - 34	**0.80 (0.73-0.87)**	0.96 (0.85-1.09)	**0.87 (0.81-0.92)**	**1.25 (1.18-1.32)**
> 34	**0.81 (0.75-0.88)**	0.97 (0.86-1.10)	**0.84 (0.79-0.89)**	0.98 (0.93-1.03)
Maternal BMI (kg/m2)				
< 20.0	Stratified	1.06 (0.89-1.25)	1.00 (0.93-1.08)	0.93 (0.86-1.00)
20.0 - 24.9		REF	REF	REF
25.0 - 29.9		**1.16 (1.04-1.31)**	**1.16 (1.10-1.22)**	**1.24 (1.18-1.30)**
> 30.0		**1.24 (1.08-1.42)**	**1.54 (1.44-1.64)**	**1.86 (1.76-1.97)**
Missing		1.04 (0.87-1.26)	0.99 (0.90-1.08)	**1.09 (1.01-1.17)**
Multiparous			**0.68 (0.65-0.72)**	
Nordic country of birth	**0.52 (0.48-0.55)**		Stratified	Stratified
Not cohabiting with father	**1.47 (1.33-1.62)**	**1.20 (1.04-1.39)**	**1.24 (1.15-1.32)**	**1.42 (1.34-1.50)**
**Maternal health characteristics**				
Tobacco use	**1.29 (1.16-1.44)**		Time dependent	**1.70 (1.61-1.80)**
Epilepsy	**1.66 (1.20-2.29)**		**1.45 (1.14-1.84)**	**1.41 (1.15-1.72)**
Asthma	**1.13 (1.00-1.27)**		**1.27 (1.18-1.37)**	**1.35 (1.27-1.43)**
Diabetes mellitus or gestational diabetes	**1.42 (1.21-1.67)**		**1.18 (1.04-1.33)**	**1.18 (1.05-1.33)**
Hypothyroidism			**1.50 (1.34-1.69)**	**1.26 (1.10-1.44)**
Autism spectrum disorder	**2.96 (2.09-4.19)**	**1.88 (1.09-3.24)**	Stratified	Stratified
ADHD/ADD	**1.34 (1.06-1.68)**		**4.10 (3.47-4.85)**	**1.61 (1.37-1.89)**
NSAID during pregnancy				**1.17 (1.07-1.28)**
PPIs during pregnancy			**1.20 (1.08-1.33)**	**1.37 (1.25-1.51)**
Prescribed antiepileptics 3 months before or during pregnancy	**1.58 (1.21-2.06)**			
Prescribed psycholeptics 3 months before or during pregnancy		**1.31 (1.09-1.58)**	**1.29 (1.19-1.41)**	**1.31 (1.21-1.41)**
Prescribed phsycoanaleptics 3 months before or during pregnancy	**1.18 (1.05-1.34)**	0.82 (0.68-1.00)	**1.45 (1.34-1.57)**	**1.33 (1.24-1.43)**
**Pregnancy / birth characteristics**				
Assisted conception				**1.19 (1.05-1.35)**
Delivery mode				
Vaginal delivery	REF	REF	REF	REF
Elective c-section	**1.23 (1.10-1.38)**	**1.32 (1.11-1.58)**	**1.29 (1.19-1.39)**	**1.13 (1.05-1.21)**
Acute c-section	**1.32 (1.20-1.45)**	**1.87 (1.65-2.12)**	**1.25 (1.17-1.33)**	**1.17 (1.10-1.25)**
Sex of child				Stratified

Boy	REF	REF	REF	
Girl	**0.61 (0.57-0.65)**	**0.76 (0.69-0.83)**	**0.31 (0.29-0.32)**	
Preterm	**2.14 (1.94-2.38)**	**4.76 (4.17-5.42)**	**1.34 (1.24-1.45)**	Time dependent
Small for gestational age	**3.24 (2.86-3.64)**	**2.00 (1.69-2.38)**	**1.52 (1.37-1.69)**	**1.51 (1.34-1.70)**
Apgar <7 at 5 minutes	**3.40 (2.94-3.94)**	Stratified	**1.73 (1.50-1.98)**	Time dependent
Small for gestational age*tobacco interaction				0.97 (0.77-1.21)

Group B streptococcus (GBS), body mass index (BMI), attention deficit/hyperactivity disorder (ADHD/ADD), nonsteroidal anti-inflammatory drug (NSAID), proton pump inhibitor (PPI), Caesarean section (C-section).

Table S2: Results from reduced multivariable Cox regression models for the four outcomes of interest as adjusted hazard ratios (aHR) and 95% confidence intervals (CI) by timing of exposure. Significant results formatted in bold.

	**Intellectual disability**		**Cerebral palsy**		**Autism spectrum disorder**		**ADHD/ADD**	
	aHR (95%CI)	n exposed	aHR (95%CI)	n exposed	aHR (95%CI)	n exposed	aHR (95%CI)	n exposed
**Pre-conception**	1.07 (0.95-1.21)	298 (7.5%)	1.06 (0.89-1.27)	137 (7.2%)	**1.09 (1.01-1.18)**	712 (7.9%)	**1.22 (1.15-1.36)**	1,067 (9.4%)
**First trimester**	1.08 (0.97-1.20)	381 (9.6%)	1.12 (0.96-1.32)	180 (9.5%)	**1.14 (1.06-1.22)**	910 (10.1%)	**1.19 (1.12-1.27)**	1,266 (11.2%)
**Second trimester**	1.08 (0.98-1.19)	476 (12.0%)	**1.30 (1.13-1.48)**	275 (14.5%)	**1.14 (1.06-1.21)**	1,118 (12.5%)	**1.23 (1.17-1.31)**	1,656 (14.6%)
**Early third trimester**	1.08 (0.90-1.29)	126 (3.2%)	**1.47 (1.18-1.84)**	86 (4.5%)	**1.23 (1.10-1.38)**	315 (3.5%)	**1.26 (1.14-1.39)**	435 (3.8%)
**Late third trimester**	1.06 (0.93-1.20)	283 (7.1%)	0.83 (0.67-1.03)	91 (4.8%)	**1.12 (1.04-1.21)**	717 (8.0%)	**1.16 (1.09-1.25)**	1,011 (8.9%)
**Possible IAP**	0.88 (0.71-1.10)	91 (2.3%)	**1.27 (1.02-1.58)**	93 (4.9%)	1.01 (0.87-1.18)	197 (2.2%)	**1.16 (1.01-1.33)**	249 (2.2%)

Attention deficit/hyperactivity disorder (ADHD/ADD), intrapartum antibiotic prophylaxis (IAP)

STROBE Checklist

STROBE Statement—Checklist of items that should be included in reports of ***cohort studies***

	Item No	Recommendation	
**Title and abstract**	1	(*a*) Indicate the study's design with a commonly used term in the title or the abstract	OK
		(*b*) Provide in the abstract an informative and balanced summary of what was done and what was found	OK
**Introduction**			
Background/rationale	2	Explain the scientific background and rationale for the investigation being reported	OK
Objectives	3	State specific objectives, including any prespecified hypotheses	OK
**Methods**			
Study design	4	Present key elements of study design early in the paper	OK
Setting	5	Describe the setting, locations, and relevant dates, including periods of recruitment, exposure, follow-up, and data collection	OK
Participants	6	(*a*) Give the eligibility criteria, and the sources and methods of selection of participants. Describe methods of follow-up	OK
		(*b*) For matched studies, give matching criteria and number of exposed and unexposed	NA
Variables	7	Clearly define all outcomes, exposures, predictors, potential confounders, and effect modifiers. Give diagnostic criteria, if applicable	OK
Data sources/ measurement	8*	For each variable of interest, give sources of data and details of methods of assessment (measurement). Describe comparability of assessment methods if there is more than one group	OK
Bias	9	Describe any efforts to address potential sources of bias	OK
Study size	10	Explain how the study size was arrived at	OK
Quantitative variables	11	Explain how quantitative variables were handled in the analyses. If applicable, describe which groupings were chosen and why	OK
Statistical methods	12	(*a*) Describe all statistical methods, including those used to control for confounding	OK
		(*b*) Describe any methods used to examine subgroups and interactions	OK
		(*c*) Explain how missing data were addressed	OK
		(*d*) If applicable, explain how loss to follow-up was addressed	OK
		(*e*) Describe any sensitivity analyses	OK
**Results**			
Participants	13*	(a) Report numbers of individuals at each stage of study—eg numbers potentially eligible, examined for eligibility, confirmed eligible, included in the study, completing follow-up, and analysed	OK
		(b) Give reasons for non-participation at each stage	OK
		(c) Consider use of a flow diagram	OK
Descriptive data	14*	(a) Give characteristics of study participants (eg demographic, clinical, social) and information on exposures and potential confounders	OK
		(b) Indicate number of participants with missing data for each variable of interest	OK
		(c) Summarise follow-up time (eg, average and total amount)	OK
Outcome data	15*	Report numbers of outcome events or summary measures over time	OK
Main results	16	(*a*) Give unadjusted estimates and, if applicable, confounder-adjusted estimates and their precision (eg, 95% confidence interval). Make clear which confounders were adjusted for and why they were included	OK
		(*b*) Report category boundaries when continuous variables were categorized	OK

		(*c*) If relevant, consider translating estimates of relative risk into absolute risk for a meaningful time period	OK
Other analyses	17	Report other analyses done—eg analyses of subgroups and interactions, and sensitivity analyses	OK
**Discussion**			
Key results	18	Summarise key results with reference to study objectives	OK
Limitations	19	Discuss limitations of the study, taking into account sources of potential bias or imprecision. Discuss both direction and magnitude of any potential bias	OK
Interpretation	20	Give a cautious overall interpretation of results considering objectives, limitations, multiplicity of analyses, results from similar studies, and other relevant evidence	OK
Generalisability	21	Discuss the generalisability (external validity) of the study results	OK
**Other information**			
Funding	22	Give the source of funding and the role of the funders for the present study and, if applicable, for the original study on which the present article is based	OK

*Give information separately for exposed and unexposed groups.

Note: An Explanation and Elaboration article discusses each checklist item and gives methodological background and published examples of transparent reporting. The STROBE checklist is best used in conjunction with this article (freely available on the Web sites of PLoS Medicine at http://www.plosmedicine.org/, Annals of Internal Medicine at http://www.annals.org/, and Epidemiology at http://www.epidem.com/). Information on the STROBE Initiative is available at http://www.strobe-statement.org.
